# EMF and the Precautionary Principle: Dolan and Rowley Respond

**DOI:** 10.1289/ehp.0901111R

**Published:** 2009-11

**Authors:** Mike Dolan, Jack Rowley

**Affiliations:** Mobile Operators Association, London, United Kingdom, E-mail: mikedolan@ukmoa.org; GSM Association, London, United Kingdom, E-mail: jrowley@gsm.org

In their letter commenting on our article (Dolan and Rowley 2009), Kundi et al. attempt to discredit us as “employees of the mobile telephone industry” and imply that we are merely advocating an industry position of support for the international radiofrequency exposure guidelines rather than addressing issues of substance regarding the application of the precautionary principle to mobile telephony. A careful reading of our article clearly demonstrates this is not the case.

With few exceptions, countries around the world have implemented the international guidelines based on many scientific reviews undertaken by experts appointed by national governments over the past decade. Kundi et al. simply ignore these reviews and their conclusions because they do not agree with them. Although Kundi et al. are entitled to hold and promulgate their own views, they should acknowledge that they are acting as advocates for lower guidelines (based on their own subjective analysis of existing scientific evidence) and they should not simply dismiss anyone who does not agree with their point of view.

Kundi et al. seem to think that the precautionary principle should be applied whenever there is some scientific doubt or uncertainty, without recognizing that its use is limited by national regulatory and legal constraints, which we addressed in our commentary (Dolan and Rowley 2009). In particular, the concerns should be plausible (World Commission on the Ethics of Scientific Knowledge and Technology 2005). As an example, the European Court of First Instance (Pfizer Animal Health SA *v.* Council of the European Union 2002) has ruled that

[A] preventive measure cannot properly be based on a purely hypothetical approach to the risk, founded on mere conjecture which has not been scientifically verified . . . . [A] preventive measure may be taken only if the risk, although the reality and extent thereof have not been fully demonstrated by conclusive scientific evidence, appears nevertheless to be adequately backed up by the scientific data available at the time when the measure was taken.

The many scientific review bodies we referred to in our article (Dolan and Rowley 2009) have not considered the existing health data on mobile telephony adequate to trigger the application of the precautionary principle.

We accept that decisions regarding application of the precautionary principle are not to be made by scientists alone because they “have neither democratic legitimacy nor political responsibilities” (Pfizer Animal Health SA *v.* Council of the European Union 2002). However, governments should not simply disregard scientific advice or adopt popularist policies and “fall prey to public fear when it is baseless” (Telstra Corporation Limited *v*. Hornsby Shire Council 2006).

In the conclusion of our commentary (Dolan and Rowley 2009), we made it clear that there are many things that governments and industry can do to better address public concern, including supporting ongoing research and conducting education and information programs for the public who, when fully informed, are better able to take their own personal precautionary measures if they wish to do so. What should be avoided is the rush to adopt measures—justified by reference to the precautionary principle—to reassure the public, because this has been shown to actually increase public concern (Weidemann and Schutz 2005).
